# Tofacitinib exposure across 2 pregnancies in alopecia universalis: Case report and literature review of Janus kinase inhibitors in pregnancy

**DOI:** 10.1016/j.jdcr.2026.05.069

**Published:** 2026-06-06

**Authors:** Abdulmajeed Alajlan, Naif Ahmed Alshehri, Sulaiman Alfouzan, Maee Barakeh, Saad Alangari, Sami Alsuwaidan

**Affiliations:** a Department of Dermatology, College of Medicine, King Saud University, Riyadh, Saudi Arabia; b Division of Dermatology, King Abdulaziz Medical City, Ministry of National Guard Health Affairs, Riyadh, Saudi Arabia; c College of Medicine, King Saud University, Riyadh, Saudi Arabia

**Keywords:** alopecia areata, alopecia universalis, case report, JAK inhibitors, lactation, literature review, outcomes, pregnancy, tofacitinib

## Introduction

Alopecia areata is a chronic autoimmune disorder characterized by nonscarring hair loss.^1^ Its unpredictable relapsing course and visible stigma impose a substantial psychosocial burden on patients and families.^1^ Janus kinase (JAK)–STAT pathway inhibition has reshaped therapy for immune-mediated disease, including alopecia areata, with clinically meaningful regrowth in moderate-to-severe cases.^2^ For patients who fail conventional modalities, JAK inhibitors can restore function and quality of life, yet pregnancy and lactation pose special challenges.^3^^,^^4^ Because systemic JAK inhibitors are small-molecule agents that are presumed to cross the placenta, and animal reproductive toxicity studies have raised concerns about adverse fetal effects, pregnancy and lactation remain important safety considerations with this drug class.^3^, ^4^, ^5^ Preclinical studies in rats and rabbits have demonstrated skeletal malformations, ventricular septal defects, reduced fetal weight, and increased pregnancy loss at exposures approximately 13- to 146-fold higher than human therapeutic levels.^4^ Given these concerns, human outcome data remain limited.^3^^,^^4^ Herein, we present the case of a 24-year-old healthy female with alopecia universalis who used tofacitinib during 2 pregnancies.

## Case presentation

A 24-year-old healthy female with alopecia universalis, who first presented to our care in 2019 with extensive alopecic patches associated with an ophiasis pattern, significantly impacting her confidence and quality of life. She had previously been managed with prednisolone 10 mg daily, methotrexate 25 mg subcutaneously once weekly. Additionally, she underwent a series of 7 intralesional steroid injections, which initially produced encouraging results. However, due to persistent new alopecic patches and frustration with incomplete responses, we explored alternative treatment options.

At age 25, given the progressive refractory nature of her condition and the failure of conventional immunosuppressants, we initiated off-label tofacitinib at 10 mg twice daily to maximize the likelihood of response after counseling and shared decision-making. Over time, she reported significant hair regrowth and disease stabilization.

At age 27, she conceived her first pregnancy while receiving tofacitinib, which she continued until approximately 6-8 weeks’ gestation. At her initial assessment after pregnancy confirmation, she was advised to discontinue tofacitinib. Although a mild relapse was observed, her first pregnancy progressed without complications. She delivered at full term via emergency cesarean section due to meconium-stained amniotic fluid. Both mother and newborn recovered well postpartum, with no congenital anomalies or neonatal complications. The infant was exclusively breastfed and followed up to 24 months without complications.

**Fig 1:**
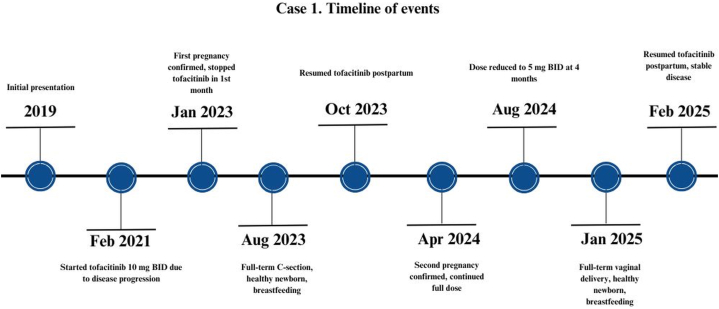
Timeline of alopecia universalis case showing tofacitinib treatment, 2 pregnancies with drug discontinuation/reduction, and healthy full-term deliveries with exclusive breastfeeding.

By the first postpartum follow-up (approximately 4 weeks after delivery), she resumed tofacitinib 10 mg twice daily with continued improvement in her condition in subsequent visits. She breastfed while on therapy without dose adjustment or timing relative to feeds. Although her second pregnancy was not initially planned, she had previously been counseled to discontinue tofacitinib at least 4 weeks prior to conception. However, she remained on her usual dose until about 20 weeks of gestation, when she reached out for guidance, and the dose was subsequently reduced to 5 mg twice daily. Throughout the remainder of her pregnancy, her disease remained stable with no major flares, and no maternal or fetal complications were observed. She again carried to full term and underwent a vacuum-assisted vaginal delivery at full term. The newborn was healthy with no reported complications and was also breastfed exclusively. A timeline of key events is shown in Fig 1.

## Discussion

Our case adds to the limited literature on tofacitinib exposure during pregnancy and lactation, documenting 2 full-term pregnancies, no congenital anomalies or major neonatal complications, and exclusive breastfeeding in both infants with no reported immediate adverse effects. To contextualize these findings, the published human pregnancy exposure data, fetal outcomes, and lactation data for systemic JAK inhibitors are summarized in Table I, Table II, Table III

**Table I:** Summary of all published studies reporting pregnancy outcomes following exposure to Janus kinase inhibitors

Study (y)	Study type	*N*/Age (y)	Condition	JAKi (dose)	Other systemic meds	Pre-con	Pregnancy	Lactation	Exposure duration GA (wk)	Infant follow-up
Baricitinib										
Bergamini (2023)^6^	Retrospective analysis of CT and PM (combined)	96/32-35	RA, AD, AA	Baricitinib 4 mg	MTX, HCQ, leflunomide, sulfasalazine, certolizumab, tocilizumab, golimumab. CMa cases co-meds: (MTX + HCQ + golimumab); (leflunomide + sulfasalazine)	✓	✓	–	1-12	NR
Costanzo (2020)^7^	Case report	1/43	RA	Baricitinib 4 mg	Methylprednisolone	✓	✓	–	1-17	9 mo
Ruxolitinib										
Urosevic (2024)^8^	Case report	1/27	Myelofibrosis	Ruxolitinib 20 mg twice daily	Interferon-α, Nadroparin, Aspirin	✓	✓	–	1-10	36 mo
Wang (2020)^9^	Case report	1/26	HLH	Ruxolitinib 5 mg/m^2^	Etoposide, IVIG, methylprednisolone	–	✓	–	≈21-22	NR
Tofacitinib										
Mitrova (2025)^10^	Case series	6/34.5	UC	Tofacitinib 5 mg twice daily or 10 mg twice daily	Vedolizumab	✓	✓	✓ (2/6)	1-12 (*n* = 3) 1-40 (*n* = 3)	3 infant were followed for 3.5, 9, and 41 mo respectively
Chaparro (2024)^11^	Case series	2/31.5	UC	Tofacitinib 10 or 5 mg twice daily	Adalimumab, Mesalamine	✓	✓	–	1-18	Neonatal period
Ernest-Suarez (2024)^12^	Case report	1/30	IBD	Tofacitinib 10 → 5 mg twice daily	None	✓	✓	✓	1-37	12 mo
Rowan (2024)^13^	Case report	1/28	UC	Tofacitinib 10 mg twice daily	5-ASA, Prednisone, Tinzaparin, Ustekinumab, Aspirin	–	✓	–	15-39	2 mo
Arzivian (2024)^14^	Case report	1/20s	UC	Tofacitinib 10 mg twice daily	Prednisolone, Vedolizumab	✓	✓	–	1-6	6 mo
Zhang (2024)^15^	Case report	1/31	SAPHO	Tofacitinib 5 mg twice daily	None	✓	✓	–	1-5	NR
Julsgaard (2024)^16^	Case report	1/39	UC	Tofacitinib 10 mg twice daily	None	–	–	✓	N/A	NR
Fernández-Sánchez (2021)^17^	Case report	1/40	PsA	Tofacitinib 5 mg twice daily	None	✓	✓	–	1-4	NR
Clowse (2016)^18^	Retrospective analysis of CT and PM (combined)	47/29	RA, psoriasis	Tofacitinib 5,10 or 15 mg twice daily or 20 mg every day	MTX, CMa case co-med: losartan 50 mg daily	✓	✓	–	1-12	NR
Upadacitinib										
Mahadevan (2024)^19^	Retrospective analysis of CT and PM (combined)	128/31.1 ± 6.2	RA, PsA, nr-axSpA, AD, UC, CD	Upadacitinib 15 mg every day; 30 mg every day (minor: 6 mg twice daily, 12 mg twice daily, 24 mg every day; none 45 mg)	MTX, OCS	–	✓	–	∼5.3 (mean)	NR
Gargiulo (2023)^20^	Case report	1/31	AD	Upadacitinib 30 mg	None	✓	✓	–	1-6	12 mo

*5-ASA*, 5-aminosalicylic acid (mesalamine); *AA*, alopecia areata; *AD*, atopic dermatitis; *CD*, Crohn disease; *CMa*, congenital malformation; *CT*, clinical trials; *GA (wk),* gestational age (weeks); *HCQ*, hydroxychloroquine; *HLH*, hemophagocytic lymphohistiocytosis; *IBD*, inflammatory bowel disease; *JAKi*, Janus kinase inhibitor; *Lac*, during lactation; *MTX*, methotrexate; *NR*, not reported; *nr-axSpA*, nonradiographic axial spondyloarthritis; *OCS*, oral corticosteroids; *PM*, postmarketing; *Pre-con*, prior to conception; *Preg*, during pregnancy; *PsA*, psoriatic arthritis; *PsO*, psoriasis; *RA*, rheumatoid arthritis; *SAPHO*, synovitis, acne, pustulosis, hyperostosis, and osteitis; *UC*, ulcerative colitis.

**Table II:** Fetal outcomes following systemic Janus kinase inhibitor exposure during pregnancy

Study (y)	Fetal outcomes					
	HNb (*N*)	CMa (*N*)	Sab (*N*)	MTe (*N*)	LFo (*N*)	Ectopic (*N*)
Baricitinib						
Bergamini (2023)^6^	25	2 (Anencephaly; developmental hip dysplasia)	14	9	35	-
Costanzo (2020)^7^	1	-	-	-	-	-
Ruxolitinib						
Urosevic (2024)^8^	1	-	-	-	-	-
Wang (2020)^9^	-	-	-	1	-	-
Tofacitinib						
Mitrova (2025)^10^	5	-	-	1	-	-
Chaparro (2024)^11^	1	1 (Polydactyly)	-	-	-	-
Ernest-Suarez (2024)^12^	1	-	-	-	-	-
Rowan (2024)^13^	1	-	-	-	-	-
Arzivian (2024)^14^	1	-	-	-	-	-
Zhang (2024)^15^	1	-	-	-	-	-
Fernández-Sánchez (2021)^17^	1	-	-	-	-	-
Clowse (2016)^18^	25	1 (Pulmonary valve stenosis)	7	8	6	-
Upadacitinib						
Mahadevan (2024)^19^	64	1 (Atrial septal defect)	37	24	-	2
Gargiulo (2023)^20^	1	-	-	-	-	-

Data are presented as number of cases.

*–*, None; *CMa*, congenital malformation; *HNb*, healthy newborn; *LFo*, lost to follow-up; *MTe*, medical termination (of pregnancy); *SAb*, spontaneous abortion.

**Table III:** Fetal outcomes following systemic tofacitinib exposure during lactation

Study (y)	*N*	Breastfeeding duration	Outcomes	Vaccination
Mitrova (2025)^10^	2	14 wk; 6 wk	Normal fetal growth and development	Received nonlive vaccination without complications
Ernest-Suarez (2024)^12^	1	12 wk	Normal fetal growth and development	Received live oral rotavirus vaccination without complications
Julsgaard (2024)^16^	1	Milk samples were collected and analyzed after intake of tofacitinib for 25 d	The highest tofacitinib milk concentration was observed 4 h after intake, and the lowest was observed 14 h after intake.	N/A

*N*, Number of cases.

## Published human pregnancy exposure data

Our review identified 289 reported pregnancies exposed to systemic JAK inhibitors, summarized in Table I. The available evidence includes case reports, case series, and retrospective analyses of clinical trial and post marketing data across several underlying diseases. The largest datasets come from retrospective safety analyses, particularly for upadacitinib and baricitinib, whereas tofacitinib is represented by multiple individual pregnancy reports.

## Fetal outcomes after in utero exposure

Fetal outcomes following systemic JAK inhibitor exposure during pregnancy are summarized in Table II. Across the published reports, the most frequently reported outcome was delivery of a healthy newborn, although spontaneous abortion, medical termination, congenital malformations, ectopic pregnancy, and loss to follow-up were also reported.

## Lactation considerations

The available human lactation data for systemic JAK inhibitors remain limited, with the most informative published data currently available for tofacitinib, as summarized in Table III. Published reports have described reassuring short-term infant outcomes in the small number of breastfed infants with maternal tofacitinib exposure. Pharmacokinetic sampling has also shown that tofacitinib is detectable in breast milk, and when exposure cannot be avoided, timing breastfeeding away from peak milk concentrations may represent a reasonable risk-reduction strategy.^10^^,^^16^

## Clinical implications

Taken together, Table I, Table II, Table III and our case suggest the following clinical implications:

• Human data on systemic JAK inhibitor exposure during pregnancy and lactation remain limited, especially in dermatology.
• Although published tofacitinib and upadacitinib outcomes do not suggest a clear excess over expected background rates, including major birth defects of approximately 2% to 4% and miscarriage in approximately 15% to 20% of clinically recognized pregnancies, evidence remains insufficient to establish safety.^11^^,^^19^^,^^21^^,^^22^
• JAK inhibitors should generally be avoided during pregnancy when possible, and patients of reproductive age should receive preconception counseling.
• For tofacitinib, the reported Tmax is approximately 0.5-1 hour and the terminal half-life is approximately 3 hours; however, product labeling recommends effective contraception during therapy and for at least 4 weeks after the last dose.^11^, ^19^, ^21^, ^22^
• Inadvertent exposure should be managed individually through counseling and shared decision-making, considering disease severity, alternatives, exposure timing and duration, and patient preferences.
• In our patient, mid-gestation dose reduction was associated with maintained disease control; however, this should not be interpreted as evidence to guide management or establish safety.
• Prospective dermatology-specific pregnancy and lactation registries with standardized reporting and longer-term infant follow-up are needed to better define maternal, fetal, and neonatal outcomes after JAK inhibitor exposure.

## Conflicts of interest

None disclosed.
